# Homozygous noncanonical splice variant in *LSM1* in two siblings with multiple congenital anomalies and global developmental delay

**DOI:** 10.1101/mcs.a004101

**Published:** 2019-06

**Authors:** Volkan Okur, Charles A. LeDuc, Edwin Guzman, Zaheer M. Valivullah, Kwame Anyane-Yeboa, Wendy K. Chung

**Affiliations:** 1 Department of Pediatrics, Columbia University, New York, New York 10032, USA;; 2 Department of Pediatrics, Naomi Berrie Diabetes Center, Columbia University, New York, New York 10032, USA;; 3 Center for Mendelian Genomics at the Broad Institute of MIT and Harvard, Cambridge, Massachusetts 02142, USA;; 4 Department of Medicine, Columbia University, New York, New York 10032, USA

**Keywords:** bicuspid aortic valve, bilateral cryptorchidism, congenital mitral stenosis, congenital strabismus, craniofacial asymmetry, cupped ear, hydronephrosis, hydroureter, inguinal hernia, intellectual disability, moderate, intermittent microsaccadic pursuits, lumbar hemivertebrae, moderate global developmental delay, penile hypospadias, perimembranous ventricular septal defect, primum atrial septal defect, triphalangeal thumb

## Abstract

Two siblings, one male and one female, ages 6 and 13 yr old, have similar clinical features of global developmental delay, multiple congenital anomalies affecting the cardiac, genitourinary, and skeletal systems, and abnormal eye movements. Whole-genome sequencing revealed a homozygous splice variant (NM_014462.3:c.231+4A>C) in *LSM1* that segregated with the phenotype in the family. LSM1 has a role in pre-mRNA splicing and degradation. Expression studies revealed absence of expression of the canonical isoform in the affected individuals. The *Lsm1* knockout mice have a partially overlapping phenotype that affects the brain, heart, and eye. To our knowledge, *LSM1* has not been associated with any human disorder; however, the tissue expression pattern, gene constraint, and the similarity of the phenotype in our patients and the knockout mice models suggest it has a role in the development of multiple organ systems in humans.

## INTRODUCTION

Birth defects/congenital malformations affect 2%–3% of all births, and genetic causes (chromosomal and monogenic disorders) account for at least 15%–20% of most anomalies (Feldkamp et al. 2017; Moorthie et al. 2018). In the last decade, exome/genome sequencing have become valuable tools to diagnose monogenic disorders, with 25%–50% diagnostic yield for multiple congenital anomalies as well as identifying new gene–disease associations (Iglesias et al. 2014; Petrikin et al. 2015; Retterer et al. 2016).

Here, we report a homozygous splice variant in *LSM1* (MIM 607281), encoding U6 snRNA-associated Sm-like protein LSm1*,* in two siblings with global developmental delay, multiple congenital anomalies affecting the heart, skeleton, and genitourinary system, and abnormal eye movements.

## RESULTS

### Clinical Presentation and Family History

This study is approved by the Institutional Review Board of Columbia University, and written informed consent was obtained for all individuals in this study. The clinical findings of the two affected siblings are summarized in Table 1.

**Table 1.** Clinical findings of individuals with homozygous splice variant in *LSM1* (NM_014462.3)

Patient ID	II.1	II.6
Gender	Female	Male
Age	13 yr old	6 yr old
Genotype	c.231 + 4A > C/c.231 + 4A > C	c.231 + 4A > C/c.231 + 4A > C
Prenatal	Bilateral hydronephrosis	Oligohydramnios Urinary tract obstruction
Intellectual disability	Yes	Yes
Developmental delay	Yes	Yes
Anthropometric measurements	*Birth* Wt: 2665 g (86%) Len: 49 cm (96%) OFC: 31.5 cm (66%) *Last visit (9 yr old)* Wt: 25.3 kg (18%) Ht: 124 cm (7%) OFC: 51 cm (25%)	*Birth* Wt: 2550 g (77%) Len: 43.5 cm (31%) OFC: 31 cm (47%) *Last visit (6.5 yr old)* Wt: 22.3 kg (25%) Ht: 115 cm (53%) OFC: n/a
Age at sitting	Delayed	27 mo
Age at walking	2.5 yr	4.5 yr
Age at talking and current speech	Delayed and 90–100-word vocabulary at age 13 yr	Delayed
Dysmorphic features	Body and facial asymmetry (left side is smaller) Brachycephaly Cupped ears Frontal bossing Hypertelorism Rounded nasal tip Micrognathia Small teeth	Flat nasal bridge Cupped right ear Excess nuchal folds
Cardiac	VSD (perimembranous) ASD PDA	Mild mitral stenosis Bicuspid (partially fused) aortic valve Mild aortic stenosis and regurgitation Dilated ascending aorta Tortuous aortic arch without obstruction Aberrant right subclavian artery
Skeletal	Vertebral anomalies Triphalangeal thumbs Fifth finger clinodactyly	Hemivertebrae (lumbar)
Genitourinary	Bilateral hydronephrosis Ovarian cyst (resolved)	Bilateral dysplastic kidneys (Transplanted) Left duplicating collecting system Hydroureter Neurogenic bladder Hypospadias VUR Bilateral inguinal hernia and cryptorchidism
Ophthalmologic	Strabismus	Mild dysfunctional saccadic pursuit
Gastrointestinal	Feeding difficulty (G-tube)	Feeding difficulty (G-tube) Constipation and diarrhea
Other	Myringotomy tubes	Sleep apnea Elevated AST and ALT (drug-induced)

(Wt) Weight, (Len) length, (OFC) occipitofrontal circumference, (Ht) height, (VSD) ventricular septal defect, (PDA) patent ductus arteriosus, (VUR) vesicoureteral reflux.

**Figure 1.**
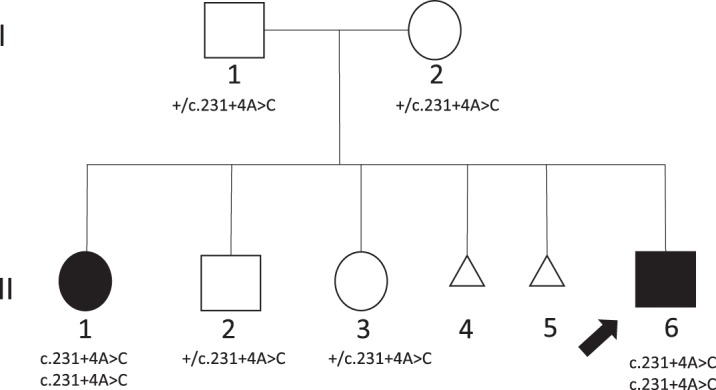
Pedigree. Affected individuals, unaffected siblings, and parents were tested for segregation analysis of noncanonical splice variant in *LSM1*. (+) Wild-type allele.

The proband (II-6; Fig. 1) is a 6-yr 7-mo-old male with multiple congenital anomalies including prenatally detected bilateral hydronephrosis, hydroureters, multiple renal cysts, hemivertebrae, and congenital heart disease. He was delivered at 34 wk of gestation because of premature cervical dilation. His birth weight was 2550 g (77th centile), length was 43.5 cm (31st centile), occipital frontal circumference (OFC) was 31 cm (47th centile), and 1- and 5-min Apgar scores were 5 and 7, respectively. He was diagnosed with posterior urethral valve obstruction, neurogenic bladder, and cystic kidney dysplasia in the newborn period. He had renal failure at birth, and nephrostomy tubes were placed. He underwent a right renal transplant at 18 mo old for end-stage renal disease with his father as the donor.

His AST and ALT were persistently elevated since initially tested at 1 mo of age. A liver biopsy at 3 yr of age showed chronic liver disease with mild portal lymphoid infiltrate, many periportal pseudo-ground-glass hepatocytes, and periportal fibrosis with early nodularity, suspected to be due to reaction to immunosuppressant medications (tacrolimus and mycophenolate mofetil) used to prevent transplant rejection.

His congenital heart disease was complex with mild mitral stenosis, a bicuspid aortic valve with partial fusion accompanied by mild aortic stenosis and regurgitation, dilation of the ascending aorta, and mildly tortuous aortic arch with an aberrant right subclavian artery but without significant obstruction. He also had mild pulmonary hypertension and sleep apnea due to pharyngeal collapse that was controlled with BiPAP and CPAP during sleep. He has mild abnormal saccadic pursuit in both eyes. He has a few minor dysmorphic facial features including flat nasal bridge, cupped right ear, and excess nuchal folds. He underwent surgeries for an inguinal hernia repair, orchiopexy, vesicostomy, and G-tube placement. His development was delayed. He sat at 27 mo, started crawling at 2.5 yr, and started walking at 4.5 yr of age. He also has speech and significant cognitive delay.

At his most recent evaluation at age 6.5 yr, his weight was 22.3 kg (25th centile) and height was 115 cm (53rd centile). He is on intermittent catheterization because of a neurogenic bladder and is fed by mouth and G-tube because of a history of poor feeding since infancy.

II-1 (Fig. 1) is the 13-yr-old similarly affected sister of the proband. She was delivered at 34 wk of gestation because of premature rupture of membranes with multiple congenital anomalies including prenatally detected bilateral hydronephrosis, congenital heart disease, and vertebral abnormalities. Her birth weight was 2700 g (86th centile), length was 49 cm (96th centile), OFC was 31.5 cm (66th centile), and 1- and 5-min Apgar scores were 3 and 7, respectively. She had a perimembranous ventricular septal defect, atrial septal defect, and patent ductus arteriosus that were surgically repaired at 7 mo of age. A unilateral ovarian cyst was detected at 2 mo of age and persisted until she was 2 yr old. She had poor feeding since birth because of severe suck and swallow incoordination and was fed via nasogastric tube in the first few months of life and switched to G-tube before a year of age. She had a Nissen fundoplication at the time of G-tube placement because of gastroesophageal reflux. She was fed by G-tube until 6 yr of age. She failed her newborn hearing screening and failed three follow-up hearing tests performed in the first two months of life secondary to serous otitis media. She had bilateral myringotomy tube placement. She has body asymmetry with the left side of the face and left upper and lower limbs smaller than the right side. She has dysmorphic features consisting of mild facial asymmetry, brachycephaly, frontal bossing, cupped ears, hypertelorism, strabismus, rounded nasal tip, micrognathia, small teeth, triphalangeal thumbs, and clinodactyly. She also has strabismus. She started walking at 2.5 yr old, but she had difficulty walking independently until she was 4.5 yr old. Her speech is delayed. She was able to speak only four-word sentences when she was 6.5 yr old, and her total vocabulary is 90–100 words as a 13 yr old. She has severe intellectual disability. MRI of the brain was normal.

Previous genetic tests performed for both siblings, including karyotype and chromosome microarray analyses, were normal. The parents are unrelated and of Ashkenazi Jewish ancestry.

### Identification of a Noncanonical Splice Variant in *LSM1* and Its Effect on Splicing

A putative splice variant in *LSM1* (NM_014462.3:c.231+4A>C) was identified by whole-genome sequencing (WGS) of the affected siblings and unaffected parents, and Sanger confirmation in all family members showed segregation of the variant in an autosomal recessive inheritance pattern (Table 2; Fig. 1). In silico splicing prediction programs predict that the variant alters splicing (Desmet et al. 2009; Gelfman et al. 2017). No other likely pathogenic sequence variants or CNVs were identified that segregated with the phenotype in the family. The c.231+4A>C variant (rs775468919) is observed in gnomAD only in the heterozygous state and predominantly in Ashkenazi Jewish individuals, with an alternate allele frequency of 0.0019 (*n* = 19 out of 10022 alleles). Submission of the gene/variant to the GeneMatcher (Sobreira et al. 2015) resulted in no match.

**Table 2.** Genomic findings

Gene	Genomic location	HGVS cDNA	HGVS protein	Zygosity	Predicted/observed effect	dbSNP ID
*LSM1*	Chr 8: 38169798 (GRCh38) Chr 8: 38027316 (GRCh37)	c.231+4A>C	Not applicable	Homozygous	Affects splicing/loss of canonical isoform expression	rs775468919

**Figure 2.**
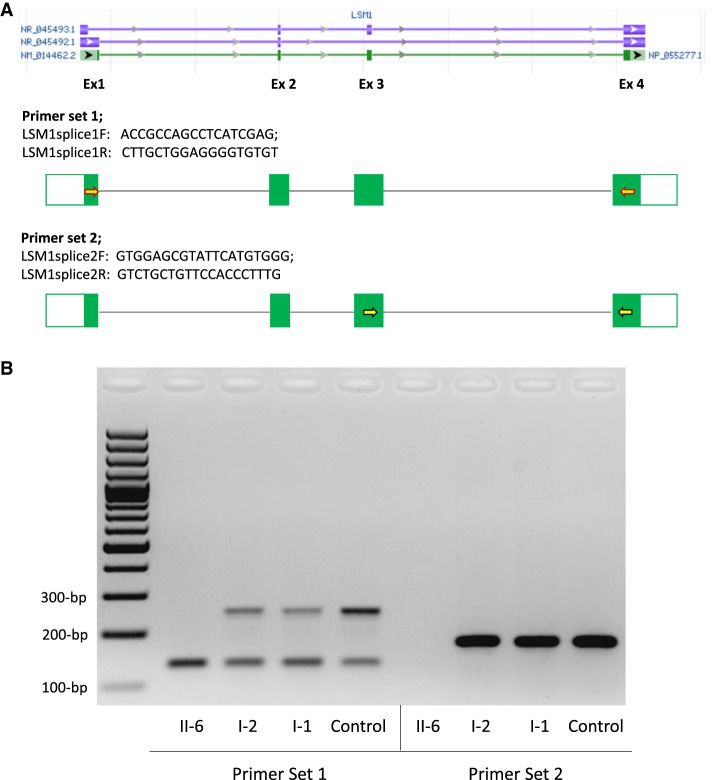
Two percent agarose-gel electrophoresis image of the cDNA studies performed with two sets of primers. Peripheral blood samples were collected from one affected individual (II-6), unaffected carrier parents (I-2 and I-1), and a control. (*A*) Two different sets of primers were designed, given the lack of exon 3 in one noncoding isoform (NR_045492.1). Forward primer of set 1 (LSM1splice1F; yellow-filled, red outlined right arrow) maps to first exons of NM_014462.2 and NR_045492.1 only. Forward primer of set 2 (LSM1splice2F; yellow-filled, black outlined right arrow) maps to exon 3 of NM_014462.2 and NR_045493.1 only. Reverse primers of both sets (LSM1splice1R and LSM1splice2R) map to exon 4 of all isoforms. Isoform accession numbers and schematic were retrieved from NCBI Entrez Gene website (Gene ID = 27257). (*B*) PCRs with primer sets 1 (*left*) and 2 (*right*) revealed no band in an affected individual (II-6) corresponding to the canonical isoform. Unaffected carrier parents (I-2 and I-1) only have one band corresponding to the canonical isoform, 250-bp band on the *left* and 176-bp band on the *right*, in addition to the 134-bp noncoding RNA transcript (NR_045492.1) product (smaller band on the *left*) in all samples run with primer set 1.

To assess the effect of c.231+4A>C on splicing, we used two different sets of primers to analyze cDNA from whole blood from the affected individual (II-6), both unaffected carrier parents (I-1 and I-2) and an unrelated noncarrier healthy individual (Control). The first primer set spanning exons 1 to 4 revealed two bands (250 and 134 bp) in samples from the parents and control, and only a single 134-bp band in the affected individual's sample (Fig. 2). With the second primer set in exons 3 and 4, there was only a single 176-bp band in each parent and the control and no band in II-6. Sequencing of the cloned PCR products confirmed that the 250-bp (primers in exons 1 and 4) and 176-bp fragments (primers in exons 3 and 4) correspond to the canonical isoform NM_014462.3, whereas the 134-bp fragment corresponds to the noncoding RNA (NR_045492.1) that lacks exon 3. Individual II-6 expressed none of the canonical isoform. The c.231+4A>C variant results in skipping of exon 3, as in NR_045492.1, and the novel transcript is predicted to produce a truncated 44-amino acid protein compared to the normal 133-amino acid protein. If translated, this protein is predicted to be missing part of the LSM domain and the rest of the carboxyl terminus of the 133-amino acid protein.

## DISCUSSION

In this study, we report a homozygous splice variant in *LSM1* (NM_014462.3:c.231+4A>C) in two siblings with global developmental delay, multiple congenital anomalies affecting the heart, skeleton, and genitourinary system, and abnormal eye movements. The variant segregates in an autosomal recessive manner with the phenotype in the family. The allele frequency for the variant is 0.0019 (1 in 250 carrier frequency) in the Ashkenazi Jewish population sample in gnomAD, and no homozygotes have been previously observed in any population databases or in our internal Ashkenazi Jewish database.

*LSM1* is located on 8p11.23 and contains four exons and encodes a 133-amino acid protein that is a member of the Sm-like (LSm) family of RNA-binding proteins that form stable heteromers that bind specifically to the 3'-terminal oligo(U) tract of U6 snRNA and play a role in pre-mRNA splicing by mediating U4/U6 snRNP formation (Achsel et al. 1999). LSM1 also plays a role in the degradation of histone mRNAs (Mullen and Marzluff 2008). In addition to the canonical isoform (NM_014462.3), there are also two noncoding RNA (ncRNA) transcripts produced via alternative splicing of unknown function (Fig. 2).

Expression studies with whole blood revealed no expression of the canonical isoform in the affected homozygous individual. The novel transcript would lack the intact LSM1 domain and the rest of the carboxyl terminus that might affect the heteromer forming with other members of LSm family—hence, the splicing and mRNA degradation efficiency. *LSM1* variants have yet to be associated with any congenital human disorder; however, its ubiquitous expression potentially accounts for the number of affected organs in our family. The *Lsm1* knockout mouse (*Lsm1^tm1b(EUCOMM)Hmgu^)* (www.mousephenotype.org) has a partially overlapping phenotype that affects the brain, heart, and eye. Abnormal neurological phenotypes (altered grip strength and abnormal behavior) are only reported in homozygous knockout mice, whereas aorta dilation and thick ventricular wall are only reported in heterozygous knockout mice. Abnormal lens morphology is reported in both homozygote and heterozygote knockout mice. Our patients have global developmental delay and congenital heart disease; however, our patients have abnormal eye function including strabismus and abnormal saccadic pursuit rather than an anatomical abnormality. Additionally, our patients have structural and functional urogenital and skeletal anomalies. Although heterozygous knockout mice exhibit a small subset of the phenotypic abnormalities, haploinsufficiency of *LSM1* in humans is tolerated according to gnomAD gene constraint metrics, and phenotypic differences between heterozygous and homozygous mice models and humans are not uncommon.

It is not yet known what the targets of LSM1 are. Genes associated with spliceosome assembly have been associated with syndromes such as mandibulofacial dysostosis, Guion–Almeida type (MIM 610536), and Nager syndrome (MIM 154400), which can include congenital heart defects, vertebral defects, genitourinary defects, and limb defects, some of which are noted in our affected individuals (for review, see Lehalle et al. 2015). The network of genes impacted downstream from the genes affecting spliceosome assembly are also not yet defined but could produce overlapping clinical symptoms.

In conclusion, we report a homozygous splice variant in *LSM1* that appears to be a founder Ashkenazi Jewish mutation that results in loss of expression of the canonical isoform in two siblings with global developmental delay, multiple congenital anomalies, and abnormal eye movements. Future families and functional studies are needed to confirm this association and to elucidate the underlying molecular and cellular mechanisms.

## METHODS

### Genomic Analyses

Whole-genome sequencing was performed on whole-blood DNA from the two affected siblings and their parents by the Genomics Platform at the Broad Institute of MIT and Harvard. PCR-free preparation of sample DNA (350 ng input at >2 ng/µL) was accomplished using HiSeq X Ten v2 chemistry (Illumina, Inc.). Libraries were sequenced to produce 151-base pair paired-end reads at mean target coverage of >30× (Table 3).

**Table 3.** Coverage parameters of whole-genome sequencing

Individual	Total reads	Total mapped reads	Average coverage	% of reads > 5×/10×	Alternate read depth
II-6	743,161,624	736,407,150	>32×	98/97	26/26
II-1	706,180,032	700,529,496	>30×	98/97	31/31
I-1	799,401,668	793,233,226	>35×	98/97	35/99
I-2	754,852,850	749,149,820	>32×	98/97	25/99

Genome sequencing data were processed through a pipeline based on Picard, using base quality score recalibration and local realignment at known insertions or deletions (indels). The BWA aligner was used for mapping reads to the human genome build GRCh38. Single-nucleotide variants (SNVs) and indels were jointly called across all samples using the Genome Analysis Toolkit (GATK) HaplotypeCaller package. Default filters were applied to SNV and indel calls using the GATK Variant Quality Score Recalibration (VQSR) approach. Annotation was performed using Variant Effect Predictor (VEP). Last, the variant call set was uploaded to *seqr* for collaborative analysis.

Variants were filtered by their population allele frequencies in Exome Sequencing Project (ESP; http://evs.gs.washington.edu/EVS/), 1000 Genomes samples (Auton et al. 2015), and Genome Aggregation Database (gnomAD) (Lek et al. 2016) using 1% and 3% thresholds for autosomal dominant and autosomal recessive inheritance models, respectively. TOPMED Freeze 5 (https://bravo.sph.umich.edu/freeze5/hg38/) was also checked for allele frequencies of variants.

Filtering based on allele frequency yielded a homozygous splice site variant in *LSM1*, homozygous missense variants in *ADGRA2*, *RAB11FIP1*, and *MPDZ*, and biallelic missense variants in *WFS1* and *VWF* that segregated with the affection status within the family (Supplemental Table 1). Given the expected high penetrance of the condition, we excluded variants with more than 10 homozygotes in gnomAD and TOPMed, which left the homozygous splice site *LSM1* variant as the only plausible candidate. The candidate variant was confirmed by Sanger sequencing in all family members.

### Expression Studies

Peripheral blood samples were collected from one affected individual (II-6), the unaffected carrier parents (I-1 and I-2), and a noncarrier healthy individual (Fig. 1). DNA was extracted using a QIAGEN Kit, and RNA was extracted using a PAXgene blood RNA kit (QIAGEN) according to the manufacturer's instructions. cDNA was reverse transcribed from the RNA using Transcriptor First Strand cDNA Synthesis kit (Roche) using mixed random hexamer primers and an anchored-oligo(dT)18 primer. The NM_014462.3:c.231+4A>C splice variant is located after coding exon 3 (exons are numbered like in ENSG00000175324). Because there is an alternatively spliced transcript lacking exon 3 (NR_045492.1), we designed two different sets of primers: one set within exons 1 (forward) and 4 (reverse) and another within exons 3 (forward) and 4 (reverse) (Fig. 2). PCR products from I-1, I-2, II-6, and Control were cloned into a TOPO vector (Invitrogen). Clones were purified by plasmid Miniprep purification kit (QIAGEN) and were sequenced using T3 and T7 universal primers (Supplemental Fig. S1).

## ADDITIONAL INFORMATION

### Data Deposition and Access

All sequence data and interpreted variants have been submitted to ClinVar (https://www.ncbi.nlm.nih.gov/clinvar/) and can be found under accession number SCV000891374. The raw sequencing data could not be deposited because of lack of patient consent.

### Ethics Statement

Written informed consent was obtained for all individuals in this study. The study is approved by the Institutional Review Board of Columbia University under protocol #AAAJ8651.

### Acknowledgments

We thank the patients and the family for participating in this study. We thank Patricia Lanzano and Jiangyuan Hu for project management, Sanger sequencing confirmations, and cDNA studies. Sequencing and data processing were provided by the Broad Institute of MIT and the Harvard Center for Mendelian Genomics (Broad CMG).

### Author Contributions

V.O. analyzed the data and drafted and critically reviewed the manuscript. C.A.L. performed expression studies and critically reviewed the manuscript. E.G. provided the clinical data and critically reviewed the manuscript. Z.M.V. analyzed the data and critically reviewed the manuscript. K.A.-Y. provided clinical data and critically reviewed the manuscript. W.K.C. conceived of the study, provided clinical data, analyzed the data, and drafted and critically reviewed the manuscript.

### Funding

This work was supported in part by grants from the National Human Genome Research Institute, the National Eye Institute, and the National Heart, Lung, and Blood Institute grant UM1 HG008900 to Daniel MacArthur and Heidi Rehm, and grants from the Simons Foundation and the JPB Foundation to W.K.C.

### Competing Interest Statement

The authors have declared no competing interest.

### Referees

Jacques S. Beckmann

Peter N. Robinson

## Supplementary Material

Supplemental Material
